# Triacylglycerol synthesis by PDAT1 in the absence of DGAT1 activity is dependent on re-acylation of LPC by LPCAT2

**DOI:** 10.1186/1471-2229-12-4

**Published:** 2012-01-10

**Authors:** Jingyu Xu, Anders S Carlsson, Tammy Francis, Meng Zhang, Travis Hoffman, Michael E Giblin, David C Taylor

**Affiliations:** 1 National Research Council of Canada, Plant Biotechnology Institute, 110 Gymnasium Place, Saskatoon, SK S7N 0W9, Canada; 2 Plant Breeding and Biotechnology, Swedish University of Agricultural Sciences, Box 101, Sundsvägen 14, 230 53 Alnarp, Sweden; 3 College of Agronomy, Northwest A & F University, No.3 Taicheng Road, Yangling, Shanxi 712100, China; 4 NRC Plant Biotechnology Institute, 110 Gymnasium Place, Saskatoon, SK S7N 0W9, Canada

**Keywords:** *dgat1 *mutant *AS11*, LPCAT1, LPCAT2, PDAT1, Oil biosynthesis Seed lines from Nottingham *Arabidopsis *Stock Centre WT (ecotype Columbia-0), *dgat1*, *AS11 *(CS3861), *A7 *(SALK_039456), *lpcat1 *(SALK_123480), *lpcat2 *(SAIL_357_H01) (all in a Columbia background)

## Abstract

**Background:**

The *Arabidopsis thaliana dgat1 *mutant, *AS11*, has an oil content which is decreased by 30%, and a strongly increased ratio of 18:3/20:1, compared to wild type. Despite lacking a functional DGAT1, *AS11 *still manages to make 70% of WT seed oil levels. Recently, it was demonstrated that in the absence of *DGAT1*, *PDAT1 *was essential for normal seed development, and is a dominant determinant in *Arabidopsis *TAG biosynthesis.

**Methods:**

Biochemical, metabolic and gene expression studies combined with genetic crossing of selected *Arabidopsis *mutants have been carried out to demonstrate the contribution of *Arabidopsis *PDAT1 and LPCAT2 in the absence of DGAT1 activity.

**Results:**

Through microarray and RT-PCR gene expression analyses of *AS11 *vs. WT mid-developing siliques, we observed consistent trends between the two methods. *FAD2 *and *FAD3 *were up-regulated and *FAE1 *down-regulated, consistent with the *AS11 *acyl phenotype. *PDAT1 *expression was up-regulated by *ca *65% while *PDAT2 *expression was up-regulated only 15%, reinforcing the dominant role of *PDAT1 *in *AS11 *TAG biosynthesis. The expression of *LPCAT2 *was up-regulated by 50-75%, while *LPCAT1 *expression was not significantly affected. *In vitro *LPCAT activity was enhanced by 75-125% in microsomal protein preparations from mid-developing *AS11 *seed *vs *WT. Co-incident homozygous knockout lines of *dgat1*/*lpcat2 *exhibited severe penalties on TAG biosynthesis, delayed plant development and seed set, even with a functional PDAT1; the double mutant *dgat1/lpcat1 *showed only marginally lower oil content than *AS11*.

**Conclusions:**

Collectively, the data strongly support that in *AS11 *it is *LPCAT2 *up-regulation which is primarily responsible for assisting in PDAT1-catalyzed TAG biosynthesis, maintaining a supply of PC as co-substrate to transfer *sn*-2 moieties to the *sn*-3 position of the enlarged *AS11 *DAG pool.

## Background

Triacylglycerols (TAGs) are the major storage lipids which accumulate in developing seeds, flower petals, anthers, pollen grains, and fruit mesocarp of a number of plant species [1,2]. TAGs are thought to be not only the major energy source for seed germination but also essential for pollen development and sexual reproduction in many plants [3,4]. In oilseeds, TAG bioassembly is catalyzed by the membrane-bound enzymes of the Kennedy pathway that operate in the endoplasmic reticulum [4]. The biosynthesis of TAGs is catalyzed by the sequential acylation of the glycerol backbone involving three acyltransferases: glycerol-3-phosphate acyltransferase (GPAT), *lyso*-phosphatidic acid acyltransferase (LPAAT) and diacylglycerol acyltransferase (DGAT). DGAT catalyses the final acylation of DAG to give TAG, which has been suggested to be the rate-limiting step in plant lipid accumulation.

In the traditional Kennedy pathway DGAT was thought to be the only enzyme that is exclusively committed to TAG biosynthesis using acyl-CoA as its acyl donor. The first *DGAT *gene was cloned from mouse and is a member of the DGAT1 family, which had high sequence similarity with sterol: acyl-CoA acyltransferase [5].

We had previously characterized an EMS-induced mutant of *Arabidopsis*, designated *AS11*, which displayed a decrease in stored seed TAG, delayed seed development, and an altered fatty acid composition [6]. We analyzed WT *vs. AS11 *lipid pools and Kennedy pathway enzyme activities in fractions isolated from green mid-developing seed, and performed parallel labeling of intact seeds at this developmental stage, with [^14 ^C] acetate. We found that compared to WT, there was an increase in all fatty acids in the DAG pool of *AS11 *seeds at mid-development, and, to a lesser extent, an associated backup of fatty acids in the PC pool. DAG was elevated from 1% in WT to 10-12% in AS11 and PC pools were elevated from about 2% in WT, to 8-12% in *AS11*. Cell-free fractions from WT and *AS11 *green seeds at mid-development were compared for their ability to incorporate [^14 ^C]-18:1-CoA into glycerolipids in the presence of G-3-P. Proportions of labeled LPA and PA formed during the incubation period were similar in WT *vs AS11*, indicating that the activities of the Kennedy pathway enzymes GPAT and LPAAT (EC 2.3.1.51) were relatively unaffected in the *AS11 *mutant. However, the proportion of labeled TAG was much lower and that of DAG was much higher in *AS11*. The TAG/DAG ratio was therefore consistently 3-5-fold lower in *AS11 *compared to WT at all developmental stages (early-, mid- and late development). Furthermore, the ratio of 18:3/20:1 dramatically increased about 7-10 fold [6].

Cumulatively, this data suggested a lesion in *DGAT1 *which was subsequently demonstrated upon cloning the mutated gene from *AS11*. There is an 81 bp in-frame insertion consisting entirely of exon 2 in the transcript from *AS11*. The exon 2 in the repeat is properly spliced, thus the alteration of the transcript does not disturb the reading frame. However, this additional exon 2 sequence in the *AS11 *transcript would result in an altered DGAT protein with a 27 amino acid insertion (^131^SHAGLFNLCVVVLIAVNSRLIIENLMK^157^) [7]. It is important to note that two other labs independently and simultaneously cloned the *A. thaliana DGAT1 *[8,9].

Studies manipulating the expression of *DGAT1 *followed: We demonstrated that expression of the *Arabidopsis DGAT1 *cDNA in a seed specific manner in the *AS11 *mutant restored wild type levels of TAG and VLCFA content. The acyl distribution, specifically, the *sn*-3 composition of the TAGs, was also restored to WT proportions. Furthermore, overexpression of the *Arabidopsis DGAT1 *in wild type plants led to an increase in seed oil content and seed weight [10]. Over the past 10 years, *DGAT1 *expression has been genetically manipulated to produce *Brassica napus *prototypes containing increased seed oil [11,12].

A second family of *DGAT *genes (*DGAT2*), first identified in the oleaginous fungus *Morteriella ramanniana*, has no sequence similarity with DGAT1 [13]. A human *DGAT2 *and plant *DGAT2s *from tung and castor were subsequently identified by Cases et al. [14], Shockey et al. [15] and Kroon et al. [16], respectively. The putative *DGAT2 *from *Arabidopsis *has been studied by several labs including ours; functional expression in yeast has not been successful, and therefore whether it is a true functioning DGAT is still in question. Notably, an *Arabidopsis dgat2 *knockout mutant has a wild-type seed oil content and fatty acid composition [17].

TAG can also be formed by an acyl-CoA-independent enzyme, phosphatidylcholine: diacylglycerol acyltransferase (PDAT), in which the transfer of an acyl group from the *sn-*2 position of PC to the *sn*-3 position of DAG yields TAG and *sn-*1 *lyso*-PC [18,19]. During the exponential growth phase in yeast, PDAT1 is a major determinant in TAG synthesis. In *Arabidopsi*s, two close homologs to the yeast *PDAT *gene have been identified: *PDAT1 *At5g13640 and *PDAT2 *At3g44830 [20]. Mhaske et al. [21] isolated and characterized a knockout mutant of *Arabidopsis *which has a T-DNA insertion in the *PDAT1 *locus At5g13640 (PDAT1, EC 2.3.1.158). Lipid analyses were conducted on this mutant to assess the contribution of PDAT1 to seed lipid biosynthesis; surprisingly, and in contrast to the situation in yeast, the oil content and composition in seeds did not show significant changes in the mutant. At the time, these results were interpreted to indicate that PDAT1 activity as encoded by At5g13640 is not a major determining factor for TAG synthesis in *Arabidopsis *seeds.

Nonetheless, because the Arabidopsis DGAT1 mutant *AS11 *shows only a 30-35% decrease in oil content [6,9], it was apparent that other enzymes must contribute to oil synthesis in the developing seed [22]. Thus, an examination of the contribution of DGAT2, PDAT2 or PDAT1 to oil deposition in an *AS11 *background was studied by performing double mutant crosses with *AS11 *[17]. While the *dgat2-ko *line has no oil phenotype, homozygous double mutants from cross of *AS11 *with *dgat2-ko *mutant showed an oil fatty acid profile similar to *AS11*. The same pattern was observed with the *pdat2-ko *mutant alone and in crosses of the *pdat2-ko *mutant with *AS11*. In contrast, while the *pdat1-ko *has no oil or fatty acid composition phenotype, crosses of the *pdat1-ko *with *AS11 *were embryo-lethal in the double homozygous condition; only heterozygous lines produced by having expression of the *pdat1 *or *dgat1 *gene only partially inhibited using RNAi, allowed an examination of the double mutants. These detailed studies resulted in the finding that DGAT1 and PDAT1 have overlapping functions in both embryo development and TAG biosynthesis in the developing seed and in pollen. When DGAT1 is compromised in *AS11*, it is PDAT1, and not DGAT2 or PDAT2, that is responsible for the remaining 65-70% of TAG which is synthesized. This finding suggested a major, perhaps dominant role of PDAT1 in this process [17].

Recently, a castor bean-specific *PDAT*, *PDAT1-2*, was cloned and found to be highly expressed in developing seeds and localized in the ER, similar to the castor FAH12 hydroxylase. Transgenic *Arabidopsis *co-expressing the castor *PDAT1-2 *and *FAH12 *showed enhanced ricinoleate accumulation to up to 25% in TAGs (compared to 17% in *FAH12 *-only transgenics) [23,24]. This study suggests that specialized PDATs may play a significant role in channeling PC-synthesized unusual fatty acids such as ricinoleic (from castor), or epoxy fatty acids (e.g. from *Vernonia galamensis*), into TAGs.

The discovery of the critical role PDAT1 has in TAG synthesis [17] suggested the importance of re-acylation of *lyso*phospholipids and especially LPC, since it is produced as a result of PDAT1 activity. We hypothesized that in a situation where the responsibility for TAG synthesis is shifted to PDAT1 such as in the *AS11 dgat1 *mutant, profound changes in activity of re-acylation enzymes would be evident. As candidates of genes coding such enzymes, we proposed the two *Arabidopsis *genes *LPLAT1 *(At1g12640) and *LPLAT2 *(At1g63050), characterized by Ståhl et al. [25] as possessing broad specificity *lyso*phospholipid acyltransferase activity. Although both of the genes to some extent could acylate a range of different *lyso*phospholipids, they were shown to have a strong preference towards LPC. For this reason we chose to name the two genes *LPCAT1 *(At1g12640) and *LPCAT2 *(At1g63050), in the current study.

Here we report the further genetic and biochemical characterization of the *AS11 dgat1 *mutant. During the course of microarray and qRT-PCR studies of *AS11 vs *WT gene expression in mid-developing siliques, we found that *LPCAT2*, encoding acyl-CoA:*lyso*phosphatidylcholine acyltransferase 2 (EC 2.3.1.23), was up-regulated while *LPCAT1 *was not affected. By a series of biochemical studies and key crosses of *AS11 *with either *lpcat1 or lpcat2 *knockout mutants, we determined that LPCAT2 (and much less so LPCAT1) is most critical for TAG synthesis in the *AS11 *mutant, primarily to maintain the PC pool for TAG assembly primarily catalyzed by PDAT1.

## Results and discussion

### Summary of the *AS11 *mutant developmental and oil phenotypes

The *AS11 *mutant line was about one week behind WT in bolting and entering the generative phase and thus, under our growing conditions, *AS11 *seed set was also delayed to four weeks post-anthesis instead of three, as typically observed in WT. For comparison, we studied another *DGAT1 *mutant, which we designated *A7*, which is a homozygous SALK line (Salk 039456) with a T-DNA insertion in the last exon of the same *DGAT1 *gene (At2g19450). *A7 *shows a developmental delay similar to that exhibited by *AS11 *(Additional file 1: Figure S1).

**Figure 1:**
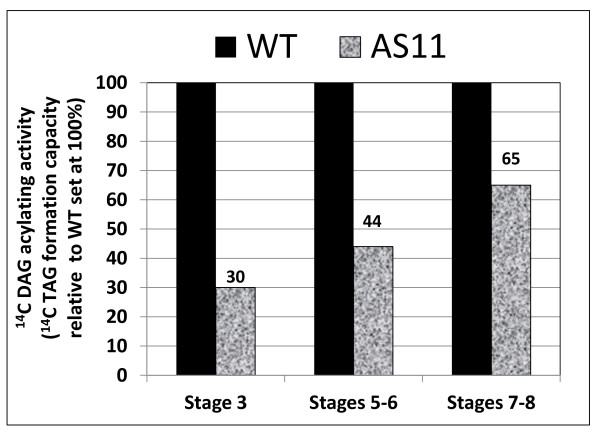
**Comparison of protein fractions from mid-developing siliques containing WT and *AS11 *embryos at stages 3, 5-6 and 7-8 (as designated in "Methods") to incorporate ^14 ^C-1,2 diolein into ^14 ^C TAG in the presence of 18:1-CoA**.

Using protein fractions prepared from WT and *AS11 *mid-developing siliques containing embryos at mid-development (Stages 6-8 as designated in the "Methods" section) we were able to determine the relative changes of TAG assembly activity in the mutant line. TAG synthesis capacity was measured in WT and *AS11 *lines with ^14^C-labeled diolein and unlabeled oleoyl-CoA as co-substrates; the ^14 ^C-labeled triolein product was measured by radio-HPLC as described previously [26]. As shown in Figure 1, by stages 7-8 there was a 35% decrease in the acylation of radiolabeled DAG in *AS11*, a finding which was strongly correlated with the *ca *30% reduction in oil content in mature *AS11 *seed [6,10].

### Heterologous expression of mutated *Arabidopsis DGAT1*s from both *A7 *and *AS11 *in Yeast Strain *H1246MATα*

**Figure 2:**
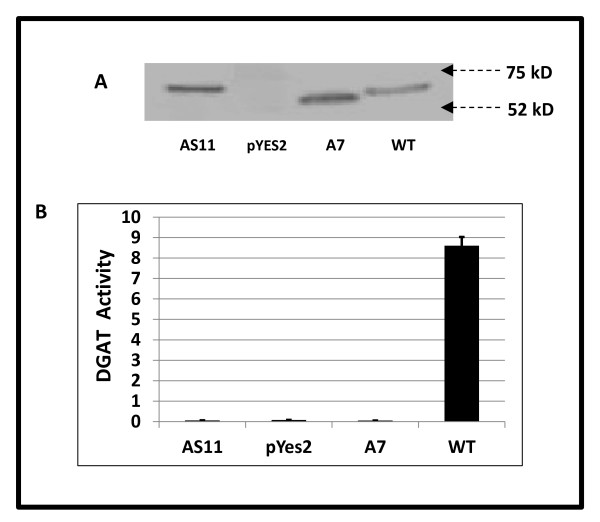
**Expression of the mutated *A7 *and *AS11 DGAT1 *and WT *DGAT1 *cDNAs in *H1246MATα *yeast cells lacking DGAT1 activity**. The insert photo (**A**) shows the western blot of the DGAT protein expressed in each lysate with the 52 kD and 75 kD M_r _marker positions indicated by the arrows. (**B**) DGAT activity in each lysate (measured as pmol ^14 ^C triolein formed · min^-1 ^· (mg protein)^-1 ^in the presence of ^14 ^C-1,2 diolein and 18:1-CoA) is reported as the average of triplicate determinations ± S.D. Only the expressed WT protein shows DGAT activity, confirming that both the *AS11 *and *A7 *DGAT1s are non-functional.

The altered fatty acid and low TAG phenotype in *AS11 *seed raised questions as to how the *AS11 *mutant still manages to make 65-70% of WT levels of seed oil. Because *AS11 *has a reduced TAG phenotype, it was essential to determine whether the DGAT1 in *AS11 *was merely mutated and exhibited reduced activity as we initially suggested [6], or whether it is, in fact, non-functional. This has not heretofore been confirmed. The importance of doing so will become apparent in the PCR assessment of the *dgat1 *transcript in both *AS11 *and in some genetic crosses performed in the current study, and discussed below. The cDNA from the *AS11 *(with a 81 bp in-frame repeat insertion of the second exon) was cloned into a yeast expression vector pYES2.1 under the control of the galactose-inducible *GAL1 *promoter, and the construct was used to transform a yeast mutant strain *H1246MATα*, which lacks all four genes, *ARE1*, *ARE2*, *DGAT1 *and *LRO1*, which were found to contribute to TAG synthesis [27]. This yeast mutant has been successfully used in studies of DGAT function wherein WT and mutated forms of plant DGAT1s have been expressed and thereby function or lack thereof in the plant inferred [28-32]. *H1246MATα *yeast cells harboring an empty pYES2.1 vector plasmid or transformed with WT *DGAT1 *cDNA were used as a negative and positive controls, respectively (Figure 2). A western blot of the microsomal membrane fractions isolated from the induced yeast *H1246MATα *cells (Figure 2A) showed that both the mutated *AS11 *and *A7 *and WT DGAT1 proteins (calculated M_r _of 62.2 kD, 53.9 kD and 58.9 kD, respectively) were stably expressed. In the pYES plasmid-only transformant, there was neither detectable DGAT1 protein, nor non-specific cross-reactivity of the antibody with non-DGAT1 proteins of a similar size. The western showed that the *AS11 *DGAT1 was slightly larger (by 27 amino acids, probably due to the exon 2 repeat) than that of WT, while for the *A7 *DGAT1, the largest possible stable transcript (truncated at the start of T-DNA insertion) encoded a protein that was significantly smaller than WT. However, as shown in Figure 2B, the *AS11 *DGAT1 could not compensate for the inability to produce TAG in this yeast quadruple mutant. Equally, when we assayed the transformed yeast microsomal protein fractions *in vitro *for DGAT activity using unlabeled oleoyl-CoA (18:1) as an acyl donor, and ^14 ^C-*sn*-1,2 diolein (18:1) as acceptor, enzyme activity was not detected in the yeast strain harboring the mutated *DGAT1 *cDNA from the *AS11 *mutant and empty control pYES2.1 vector, but was found in the positive WT control. This indicated, perhaps not unexpectedly, but for the first time, that the mutated dgat1 from *AS11 *is non-functional. Equally, the truncated DGAT1 from the *A7 *mutant (SALK-039456) was shown to be non-functional when expressed in *H1246MATα *cells (Figure 2B).

Thus our results strongly support that DGAT1 is not functioning in the developing seed of the *AS11 *mutant line. Clearly, the (radiolabeled) TAG formation observed in protein fractions from *AS11 *was coming from another path and not via reduced DGAT1 catalysis.

Given the importance of PDAT1 in oil biosynthesis in *Arabidopsis *as we earlier defined [17], and combining this new information with the ^14^C TAG biosynthesis results in assays of WT *vs AS11 *protein fractions reported above, it raised the question that, if not from DGAT1, DGAT2 nor PDAT2, what biochemical steps besides PDAT1 may be critical for TAG biosynthesis?

### AS11 microarray and qRT-PCR analyses

**Figure 3:**
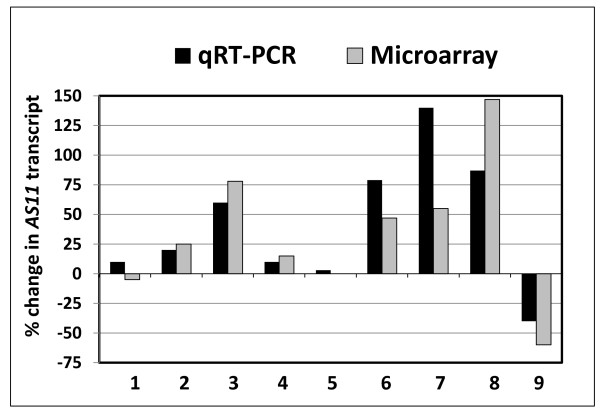
**Comparison of change in expression of lipid metabolism genes selected from microarray data and from semi-quantitative RT-PCR data, each presented as % change in *AS11 *transcript (relative to WT)**. Both assessments were conducted using the same triplicate biological RNA samples isolated from mid-developing siliques containing WT and *AS11 *embryos at stages 7-8. Genes are numbered 1-9 and named by their acronym and AGI number as follows: (1) *DGAT1 *(At2g19450); (2) *DGAT2 *(At3g51520); (3) *PDAT1 *(At5g13640); (4) PDAT2 (At3g44830); (5) *LPCAT1 *(At1g12640); (6) *LPCAT2 *(At1g63050); (7) *FAD2 *(At3g12120); (8) *FAD3 *(At2g29980); (9) *FAE1 *(At4g34250).

These cumulative findings prompted us to examine a broader inventory of gene transcripts/encoded proteins that may be involved in regulating lipid biosynthesis in *AS11 *when DGAT1 activity is compromised, compared to their corresponding expression pattern in WT. Thus we performed a microarray analysis of gene expression in mid-developing seeds of mutant *AS11 *and WT *Arabidopsis*. Based on selected probable lipid assembly-related transcripts [33] differentially expressed through the microarray study, we complemented this with a semi-quantitative qRT-PCR analysis and compared the % change in *AS11 *transcript relative to WT, for each. While neither method is truly quantitative, the *qualitative trends *in each study were highly consistent (Figure 3).

Some general observations from these combined gene expression studies follow: In *AS11*, *FAE1 *is down-regulated, *FADs 2 *&*3 *are up-regulated; this is consistent with the *AS11 *acyl composition profile (reduced 20:1 and elevated 18:3). *DGAT1 *expression was not significantly affected. Interestingly, *LPCAT2 *was up-regulated by an average of 65%, while *LPCAT1 *expression was indifferent. *PDAT2 *was only marginally affected, but *PDAT1 *expression was up-regulated by an average of 62% compared to WT.

Given the critical role of PDAT1 in embryo development and TAG deposition in both pollen and seeds [17], we were interested in the relative differences in *LPCAT1 *and *LPCAT2 *expression between the *AS11 *mutant and WT. We hypothesized that an acyl-CoA-dependent LPCAT may be critical to maintain the PC pool as one of the co-substrates for PDAT1-catalyzed TAG synthesis, particularly in the absence of a functional DGAT1, and performed a series of genetic and metabolic studies to examine these relationships.

### RT-PCR Characterization of the *lpcat1*, *lpcat2 *and *dgat1 *(*AS11*) mutants

**Figure 4:**
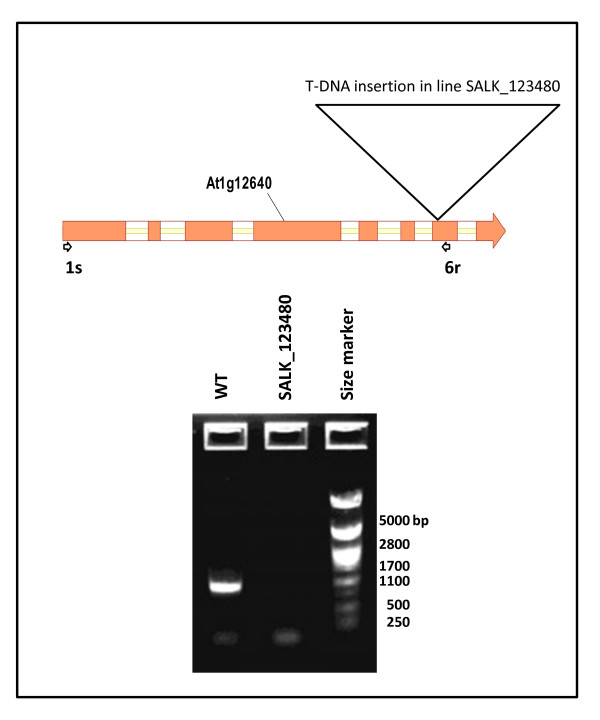
***LPCAT1 *expression in Col WT and *lpcat1 *T-DNA insertion knockout mutant Salk_123480**. RT-PCR was conducted as described in "Methods" and show that the transcript for *LPCAT1 *is absent in the Salk_123480 mutant. The map shows the position of the T-DNA insertion in *LPCAT1 *gene.

**Figure 5:**
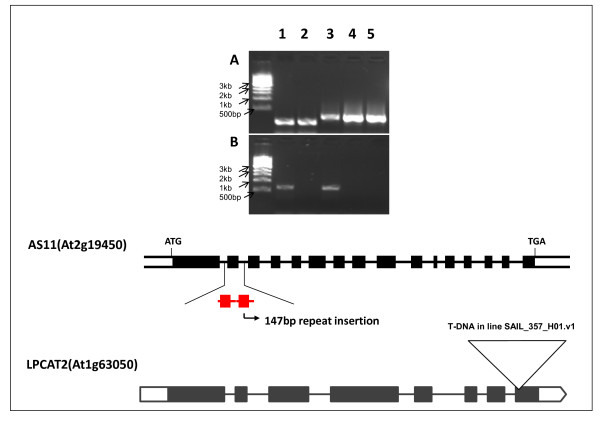
**(**A**) *DGAT1 *expression Lane 1: WT; Lane 2 *lpcat2 *H single mutant, Lane 3-*AS11 dgat1 *single mutant; Lane 4 *dgat1/lpcat2 *H/H double mutant line #6-3-10-19; Lane 5 *dgat1/lpcat2 *H/H double mutant line #6-3-20-1**. (**B**) *LPCAT2 *expression: Lane 1: WT; Lane 2 *lpcat2 *H single mutant SAIL_357_H01, Lane 3-*AS11 dgat1 *single mutant; Lane 4 *dgat1/lpcat2 *H/H double mutant line #6-3-10-19; Lane 5 *dgat1/lpcat2 *H/H double mutant line #6-3-20-1. RT-PCR was conducted using *DGAT1*-specific primers (**A**) and *LPCAT2*-specific primers (**B**) as described in "Methods". The maps show the position of 147 bp repeat insertion in the *AS11 DGAT1 *gene and the T-DNA insertion site in the *LPCAT2 *gene.

In addition to the WT and *AS11 *lines, we selected accession lines with putative mutations in *LPCAT1 *and *LPCAT2*, and characterized each of the individual mutants using RT-PCR to confirm the transcript phenotype of each line. As shown in Figure 4, in the putative *lpcat1 *mutant the *ca *1100 bp transcript was absent, but it was amplified in the control (WT) sample, indicating that the mutant At1g12640 is indeed a knockout for the *LPCAT1 *gene. Similarly as shown in Figure 5B, lane 2, the band amplified for the *LPCAT2 *transcript is absent in the putative *lpcat2 *mutant, but present in the WT and *AS11 *mutant (lanes 1 and 3, respectively) confirming that line At1g63050 is a knockout for the *LPCAT2 *gene. As expected, both the WT and the *lpcat2 *mutant samples are positive for the WT *DGAT1 *amplicon (Figure 5A, lanes 1 and 3, respectively). As shown in Figure 5A, lane 3, the *AS11 *mutant *dgat1 *transcript is present, encoding the in-frame exon 2, 27-amino acid repeat, the amplified fragment being 81 bp larger than that found in the WT and in the *lpcat2 *mutant. However, as demonstrated in Figure 2 and discussed above, though present, we have very strong evidence from yeast expression studies that the mutant dgat1 encoded by this transcript is non-functional.

### Metabolic studies

**Figure 6:**
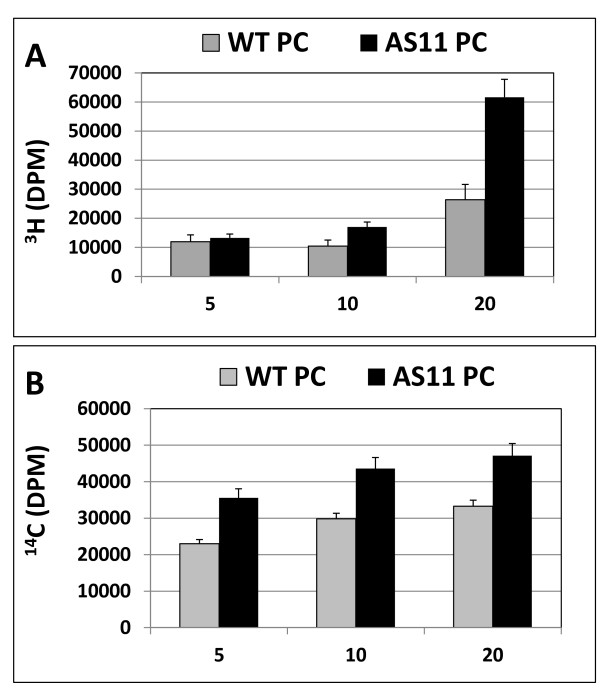
**Relative LPCAT activity in protein fractions from mid-developing siliques containing WT and *AS11 *embryos at stages 7-8 (as designated in Methods")**. LPCAT activity was monitored by appearance of (**A**) ^3 ^H and (**B**) ^14 ^C label in PC, during a 20 min incubation of WT or *AS11 *protein in the presence of L-α-palmitoyl- [1-^3 ^H methyl] *lyso*-3-phosphatidylcholine and [1-^14 ^C] 18:1-CoA. The data represent the average of two experimental replicates ± S.D. While absolute values differed, similar trends were observed in a second independent biological replication.

We performed *in vitro *LPCAT assays in a time course incubation of protein fractions from *AS11 *and WT mid-developing seed, in the presence of *sn*-1 palmitoyl-^3 ^H LPC + ^14 ^C-18:1-CoA and followed the ^3 ^H and ^14 ^C labeling patterns in PC. As shown in Figure 6A the ^3 ^H accumulation pattern indicated that tritiated PC was rapidly synthesized from ^3 ^H LPC at a rate that was 3.5-fold higher in *AS11 *than WT within 20 min. Based on the proportion of *sn*-2 ^14 ^C oleoyl moieties incorporated into PC, the LPCAT activity was consistently 40-60% higher in *AS11 *at all time points (Figure 6B). Equally, the proportion of ^3^H in LPC concomitantly decreased in *AS11 *relative to WT over this period (data not shown). This indicated that in *AS11*, the LPCAT activity was strongly enhanced relative to WT.

**Figure 7:**
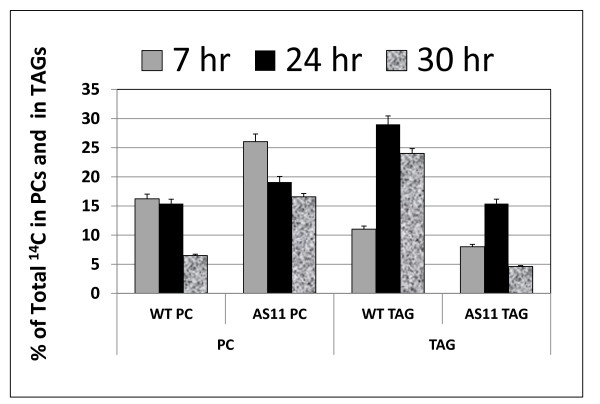
**Relative incorporation of ^14 ^C into PC and into TAG in WT *vs AS11 *following an *in vivo *feeding experiment in which severed bolted stems containing mid-developing siliques were supplied with 1-^14 ^C sodium acetate over a 30 h time course**. Seeds were harvested at each time point and seed lipids isolated and quantified as described in Methods. Results are the average of triplicate experimental determinations ± S.D.

We also performed an *in vivo *feeding study wherein we supplied ^14 ^C acetate to bolted stems containing pods with mid-developing seeds of both *AS11 *and WT plants, and then analyzed the label patterns in various lipid fractions in the mid-developing seeds after a chase period of 7, 24 and 30 h. Two major differences were immediately obvious (Figure 7): *AS11 *showed a higher relative incorporation of ^14 ^C into PC and a lower relative incorporation of ^14 ^C into TAG over the time course. These trends were entirely consistent with an elevated LPCAT activity and reduced TAG synthesis in *AS11 *as shown in the *in vitro *LPCAT (Figure 6) and DGAT1 (Figure 1) assays.

Based on these trends we were confident that in addition to DGAT1 and PDAT1, LPCAT plays a significant role in TAG biosynthesis, and postulated that it is important not only for membrane development and acyl turnover therein, but also to replenish the supply of PC for PDAT1-catalyzed TAG biosynthesis in the developing seed. To resolve this question we needed to study what occurs when both *DGAT1 *and *LPCAT *expression are co-disrupted.

### Genetic crosses

It had previously been shown, following expression in a yeast *lca1Δ *mutant background, that both *Arabidopsis LPCAT1 *(At1g12640) and *LPCAT2 *(At1g63050) are functional LPCATs. In the presence of *sn*-1 [^14 ^C] 16:0-LPC and 18:1-CoA (the same combination of substrates used in the current study), expressed LPCAT1 and LPCAT2 proteins had activities of 12 nmol/min/mg/mg protein and 18 nmol/min/mg protein, respectively [34].

As confirmed by RT-PCR (discussed above), *lpact1 *(At1g12640) and *lpcat2 *(At1g63050) are T-DNA insertion knockouts for their respective genes, while *AS11 *encodes a mutated and non-functional (Figure 2) dgat1. To examine the relationship between these two LPCATs and DGAT1 in TAG biosynthesis, we performed crosses of the *AS11 dgat1 *with the *lpcat1 *or the *lpcat2 *T-DNA insertion knock-out mutants and characterized the hemizygous/homozygous and double knockout seed oil profiles.

#### dgat1 × lpcat1

**Figure 8:**
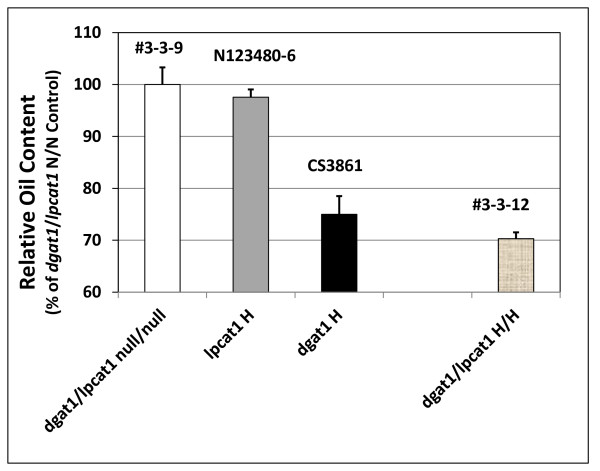
**Relative oil content in F_2 _seeds from crosses of *lpcat1 *and *AS11 *mutants**. Line numbers are designated as listed in Additional file 2: Table S3, with genotypes designated on the × axis. *dgat1*/*lpcat1 *null for both mutations # 3-3-9; *lpcat1 *homozygous mutant N123480-6; *AS11 dgat1 *homozygous mutant CS3861; *dgat1*/*lpcat1 *homozygous for both mutations #3-3-12. The values are reported relative to the double null controls set at 100%. Values are the average of triplicate determinations ± S.D.

The *lpcat1 *mutant is devoid of any significant oil phenotype compared to its null segregant or to WT (Figure 8). Crosses of *AS11 (dgat1) *with the *lpcat1 *yielded progeny homozygous for both mutations. The double homozygous mutant showed normal plant development and seed set. Zhang et al. [17] showed that without DGAT1, the PDAT1 route contributed 75% oil in AS11; thus even without combined contributions from [DGAT1 + LPCAT1], the PDAT1 route could still provide up to 70% oil in the developing *AS11 *seeds. In other words, the co-incident loss of LPCAT1 with DGAT1 reduced the capacity for PDAT1-catalyzed oil synthesis by only 5%.

**Figure 9:**
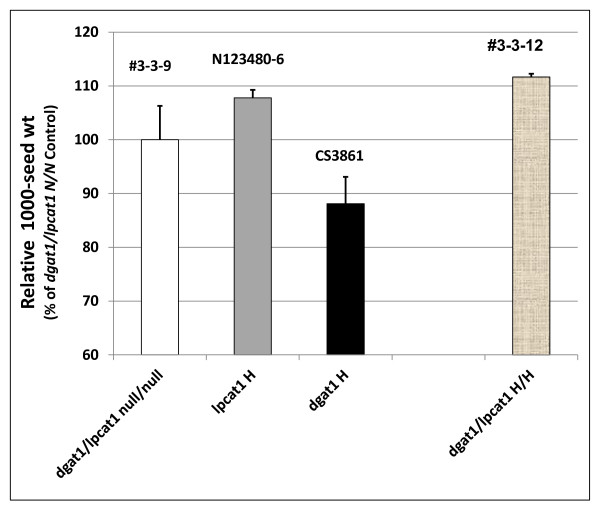
**Relative 1000-seed weight in F_2 _seeds from crosses of *lpcat1 *and *AS11 *mutants**. Line numbers are designated as listed in Additional file 2: Table S3, with genotypes designated on the × axis. *dgat1*/*lpcat1 *null for both mutations # 3-3-9; *lpcat1 *homozygous mutant N123480-6; *AS11 dgat1 *homozygous mutant CS3861; *dgat1*/*lpcat1 *homozygous for both mutations #3-3-12. The values are reported relative to the double null controls set at 100% and are the average of triplicate determinations ± S.D.

While oil content in the *dgat1*/*lpcat1 *H/H line is reduced, it is interesting to note that with respect to seed weight, there is a significant increase (by *ca *6%) relative to the *dgat1/lpcat1 *null/null control (Figure 9). Thus, while there was no penalty in this regard, further study is needed to ascertain which components of the seed e.g. protein or cellulosic biomass of the seed coat) account for this observation.

**Figure 10:**
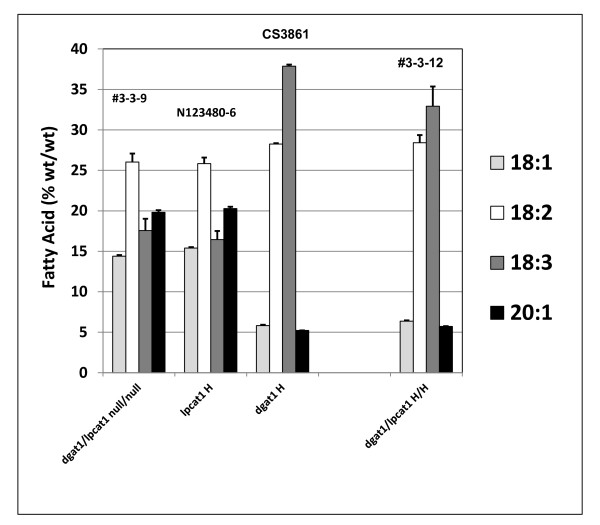
**Compositional changes in key fatty acids in TAGs from F_2 _seeds of crosses of *lpcat1 *and *AS11 *mutants**. Line numbers are designated as listed in Additional file 2: Table S3, with genotypes designated on the × axis. *dgat1*/*lpcat1 *null for both mutations # 3-3-9; *lpcat1 *homozygous mutant N123480-6; *AS11 dgat1 *homozygous mutant CS3861; *dgat1*/*lpcat1 *homozygous for both mutations #3-3-12. The values are reported as % (wt/wt) and are the average of triplicate determinations ± S.D.

The fatty acid profile from the *lpcat1 *mutant is identical to that of its null segregant (Figure 10). The *AS11(dgat1) *x *lpcat1 *double knockout lines show an *AS11*-like profile (low 18:1 and 20:1; high 18:3); the 18:3 proportion is about 2-5% lower than in *AS11 *alone. The latter is not inconsistent with the fact that LPCATs are involved in the shuttling of 18:1 moieties to the PC backbone for desaturation by FADs 2 and 3.

#### dgat1 × lpcat2

**Figure 11:**
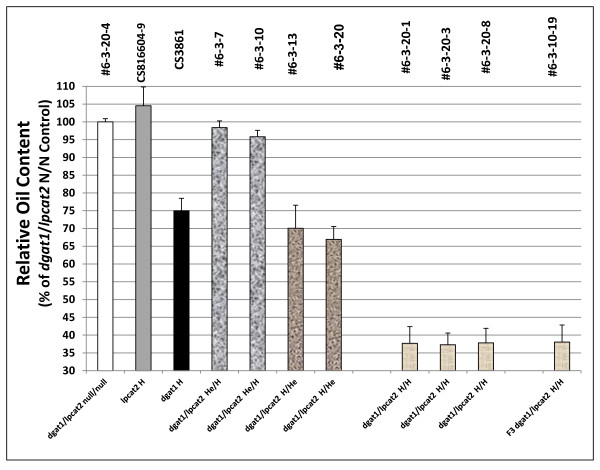
**Relative oil content in F_2 _seeds from crosses of *lpcat2 *and *AS11 *mutants**. Line numbers are designated as listed in Additional file 2: Table S3, with genotypes designated on the × axis. *dgat1*/*lpcat2 *null for both mutations # 6-3-20-4; *lpcat2 *homozygous mutant CS816604-9; *AS11 dgat1 *homozygous mutant CS3861; *dgat1*/*lpcat2 *He/H #6-3-7 and # 6-3-10; *dgat1*/*lpcat2 *H/He # 6-3-13 and # 6-3-20; *dgat1*/*lpcat2 *homozygous for both mutations #6-3-20-1, # 6-3-20-3 and # 6-3-20-8; F_3 _*dgat1*/*lpcat2 *homozygous for both mutations # 6-3-10-19. The values are reported relative to the double null controls set at 100% and are the average of triplicate determinations ± S.D.

**Figure 12:**
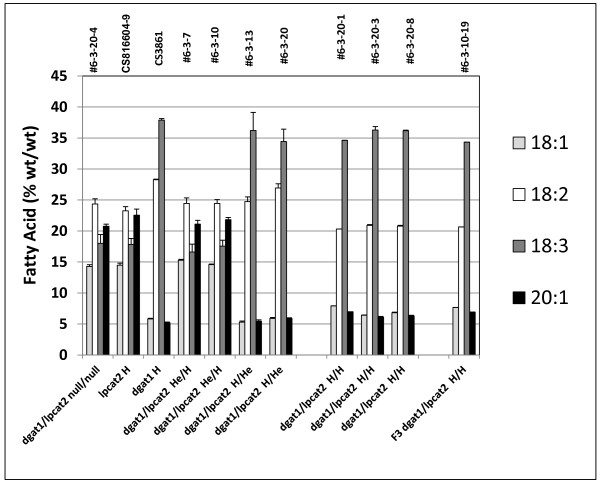
**Compositional changes in key fatty acids in TAGs from F_2 _seeds of crosses of *lpcat2 *and *AS11 *mutants**. Line numbers are designated as listed in Additional file 2: Table S3, with genotypes designated on the × axis. *dgat1*/*lpcat2 *null for both mutations # 6-3-20-4; *lpcat2 *homozygous mutant CS816604-9; *AS11 dgat1 *homozygous mutant CS3861; *dgat1*/*lpcat2 *He/H #6-3-7 and # 6-3-10; dgat1/lpcat2 H/He # 6-3-13 and # 6-3-20; dgat1/lpcat2 homozygous for both mutations #6-3-20-1, # 6-3-20-3 and # 6-3-20-8; F3 dgat1/lpcat2 homozygous for both mutations # 6-3-10-19. The values are reported as % (wt/wt) and are the average of triplicate determinations ± S.D.

The *lpcat2 *mutant does not have a significant oil phenotype compared to its null segregant-i.e. it is similar to WT (Figure 11). The fatty acid composition is essentially identical to WT and to that exhibited by the *LPCAT2 *null segregant (Figure 12). When we performed the *AS11(dgat1) *x *lpcat2 *crosses, the *dgat1*/*lpcat2 *He/H shows only a small reduction in oil content which suggests that when DGAT1 is partially expressed (He), and LPCAT2 is knocked out (H), there is a reduced capacity for compensation in TAG biosynthesis, probably because PDAT is insufficiently "fueled" with its co-substrate, PC. In these lines there is a severe penalty on seed development; there are many gaps in developing siliques due to non-fertilized ovules (Additional file 1: Figure S2A,B).

**Figure 13:**
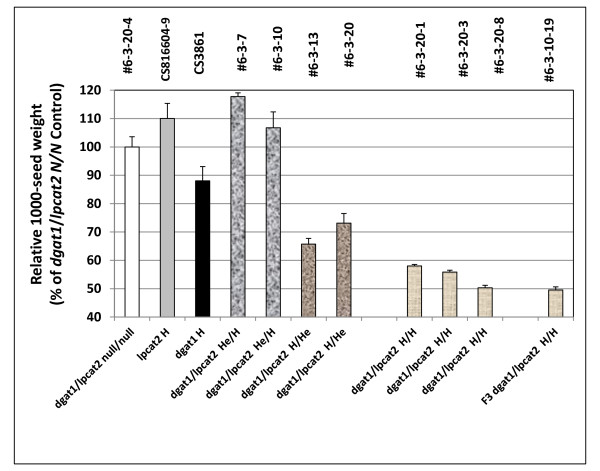
**Relative 1000-seed weight in F_2 _seeds from crosses of *lpcat2 *and *AS11 *mutants**. Line numbers are designated as listed in Additional file 2: Table S3, with genotypes designated on the × axis. *dgat1*/*lpcat2 *null for both mutations # 6-3-20-4; *lpcat2 *homozygous mutant CS816604-9; *AS11 dgat1 *homozygous mutant CS3861; *dgat1*/*lpcat2 *He/H #6-3-7 and # 6-3-10; *dgat1*/*lpcat2 *H/He # 6-3-13 and # 6-3-20; *dgat1*/*lpcat2 *homozygous for both mutations #6-3-20-1, # 6-3-20-3 and # 6-3-20-8; F_3 _*dgat1*/*lpcat2 *homozygous for both mutations # 6-3-10-19. The values are reported relative to the double null controls set at 100%, and are the average of triplicate determinations ± S.D.

Relative to the *dgat1*/*lpcat2 *double null, in the *lpcat2 *H and *dgat1*/*lpcat2 *He/H lines, seed weight is increased by about 10% (Figure 13). However, the *dgat1/lpcat2 *H/H lines show an strong decrease in seed weight even beyond that observed in the *dgat1 *mutant alone or when the double mutant genotype has *dgat1 *in the homozygous condition, Taken together with the seed weight increases cited above for the *lpcat1 *mutant studies, these findings make it very clear that both the *lpcat1 *or *lpcat2 *mutations have a complicated effect on seed weight which may or may not be related to changes in oil content.

The acyl composition of the *dgat1*/*lpcat2 *He/H lines was again essentially like the *LPCAT2 *NS (Figure 12). In contrast, the *dgat1*/*lpcat2 *H/He lines have an *AS11*-like reduction in oil content which is about 70% of that found in the WT and the *LPCAT2 *null segregant (Figure 11). Interestingly, there is no developmental penalty in this heterozygous combination and seed set is normal as in the *AS11 *background, provided expression of *LPCAT2 *is in the heterozygous state (Additional file 1: Figure S3). Not unexpectedly, the acyl composition of the oil in these lines is consistent with the *AS11 *profile (Figure 12).

**Figure 14:**
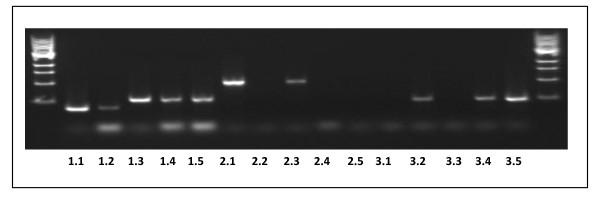
**PCR confirmation of the genetic makeup of lpcat2 × dgat1 lines**. **Samples 1.1-1.5 **(from WT, *lpcat2*, *dgat1*, and *dgat1*/*lpcat2 *H/H lines # 6-3-10-19 and # 6-3-20-1, respectively) were amplified using DGAT1 primers (Additional file 2: Table S2). Samples from *dgat1*, **(1.3)**, *dgat1*/*lpcat2 *H/H lines # 6-3-10-19 **(1.4) **and # 6-3-20-1 **(1.5)**, all have a 147 bp insert indicating they are homozygous for the insertion mutation resulting in a non-functional DGAT1 (as confirmed in Figure 2, above), while the WT **(1.1) **and *lpcat2 ***(1.2) **samples do not. T-DNA screening was performed on the samples of the same lines to test for the presence of *LPCAT2*. **Samples 2.1-2.5 **(same order as above) were amplified using SAIL_357_H01LP and SAIL_357_H01RP primers (Additional file 2: Table S2) and **samples 3.1-3.5 **(same order as above) were amplified using SAIL_357_H01RP and SAIL_LB2 primers (Additional file 2: Table S2). Collectively, sets 2.1-2.5 and 3.1-3.4 show that the *lpcat2*, and *dgat1*/*lpcat2 *H/H lines # 6-3-10-19 and # 6-3-20-1 samples have a homozygous knockout of the *LPCAT2 *gene.

The results were quite different in the *AS11 (dgat1) *x *lpcat2 *double homozygous mutant. Shown in Figure 14 is a PCR confirmation of the genotype of putative H/H lines # 6-3-10-19, # 6-3-20-1 compared to WT, *AS11 *and *lpcat2 *lines. AS11, and *dgat1/lpcat2 *H/H lines # 6-3-10-19 and # 6-3-20-1 have the expected 147 bp insert indicating they are homozygous for the insertion mutation resulting in a non-functional DGAT1 (as shown in Figure 2, above), while the WT and *lpcat2 *samples do not. T-DNA screening was done on the samples of the same lines to test for the presence of *LPCAT2*. Samples were amplified using [left primer + right primer] or with [right primer + left border 2] primer. The *lpcat2 *and the *dgat1/lpcat2 *H/H lines # 6-3-10-19 and # 6-3-20-1 have a homozygous knockout of the *LPCAT2 *gene. qRT-PCR also confirmed the transcript profile in these two lines for both *dgat1 *(Figure 5A, lanes 4 and 5) and *lpcat2*, (Figure 5B, lanes 4 and 5).). Biochemically, in the double homozygous mutant (*dgat1*/*lpcat2 *H/H) there is a severe penalty on TAG synthesis; there is a 65% reduction in oil content relative to WT: Mature seeds have only about 30% oil content relative to the WT and the LPCAT2 null segregant proportions (Figure 11). Without DGAT1 (*AS11*), recent studies suggested that PDAT1 catalyzes the synthesis of essentially all the oil which accumulates at 70-75% of that found in WT [17]. Without both LPCAT2 and DGAT1, PDAT1 is able to synthesize only about 35% of WT oil levels. Thus, the loss of LPCAT2 strongly reduces the capacity of PDAT1 to synthesize oil. This suggests a strong link between PDAT1 and its supply of PC as a co-substrate in TAG synthesis; clearly LPCAT2 plays a strong role in supplying this PC. When the DGAT1 mutation is homozygous, the *AS11 *acyl profile dominates when the LPCAT2 mutation is in the He condition (Figure 12). However, in both the *lpcat2 *homozygous mutant and in the double mutant *dgat1*/*lpcat2 *H/H lines, the 20:1 and 18:1 are slightly *increased *while 18:2 is *decreased *(Figure 12). It is significant to note that Shen et al. [34] demonstrated that an *Arabisdopsis *line transformed with an *LPCAT1-LPCAT2 *double RNAi construct, partially repressing the expression of both genes, also exhibited a 3% *increase *in the proportion of 20:1, a 1.5% *increase *in 18:1 and a concomitant 3% *decrease *in 18:2 in seed TAGs compared to WT. While the current phenotype is difficult to interpret without further study, these findings will be important in future analyses of LPCATs in terms of substrate selectivity and potential channeling of fatty acids into oil.

**Figure 15:**
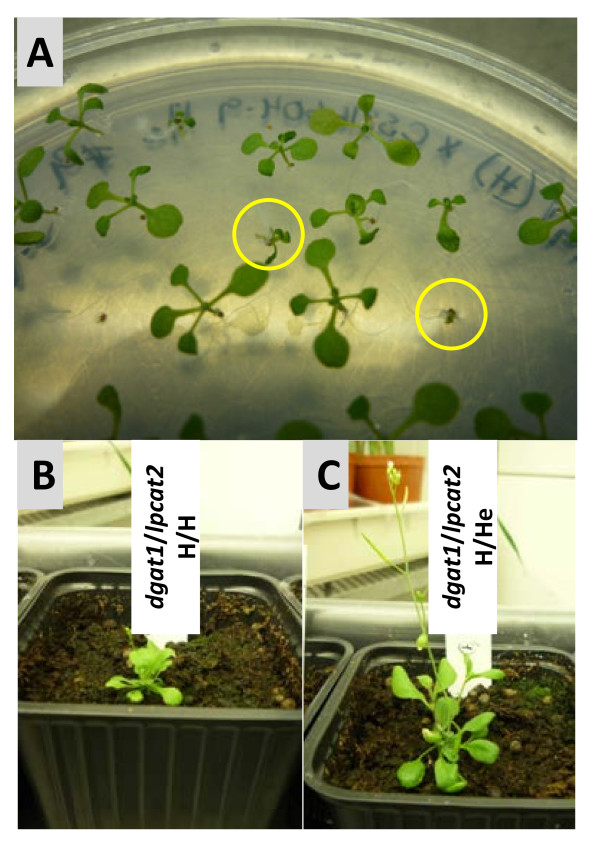
**(A) Segregating populations from crosses of *dgat1 *with *lpcat2***. All lines are homozygous for the *dgat1 *mutation and homozygous, heterozygous or null for *lpcat2*. The *dgat1*/*lpcat2 *H/H seedlings are circled, showing slow development. **(B) **Once transferred to soil, bolting is delayed in the *dgat1*/*lpcat2 *H/H seedlings. In contrast **(C) ***dgat1*/*lpcat2 *H/He seedlings develop and bolt normally.

Plant growth and development is also delayed in the double homozygous mutant (*dgat1*/*lpcat2 *H/H), as it is in *AS11*: the seedlings are small and slow to develop (Figure 15A). Once transferred to soil, bolting to enter the generative phase is delayed in *dgat1*/*lpcat2 *H/H (Figure 15B), but is normal in *dgat1*/*lpcat2 *H/He (Figure 15C). Seed development is poor, as in the *dgat1*/*lpcat2 *He/H lines; there are many non-fertilized ovules in developing siliques resulting in seed which is visibly reduced compared to that normally observed in WT or *LPCAT2 *null segregant (NS). Thus, it is clear that some level of *LPCAT2 *expression is necessary for normal seed development, especially in an *AS11 *background. However, future investigations of this preliminary observation, including scoring of developmental phenotypes and a microscopic study of pollen viability, will be required to confirm a disturbed pollen functionality as a result of the double mutation in the *dgat1*/*lpcat2 *H/H lines.

Summarizing the mutant crossing experiments, in *lpcat2 × dgat1*, oil synthesis, vegetative growth and seed set are severely penalized. Compared to the relative oil content in *dgat1 *alone (75% of WT), there is only a 5% further relative decrease in *dgat1/lpcat1 *oil (70% of WT) which suggests that LPCAT2 is the major determinant and can almost fully compensate for the loss of LPCAT1. In contrast, in the co-incident absence of LPCAT2 and DGAT1 (*lpcat2 × dgat1*), relative oil content drops to 30% of WT, which suggests that LPCAT1 can only partially compensate for the loss of LPCAT2 with respect to oil. Metabolic and microarray studies in *AS11 *alone support this hypothesis as *LPCAT2 *expression and LPCAT activity are up-regulated in the absence of DGAT1. In addition, the acyl profiles indicate that any altered oil composition phenotype observed in the *lpcat2 *x *dgat1 *is predominantly influenced by the *dgat1 *lesion. Collectively, the data from the crosses of *AS11 *with *lpcat*1 or *lpcat2 *mutants strongly suggest that in an *AS11 *background, while both LPCAT2 and LPCAT1 can contribute to PDAT1-catalyzed TAG synthesis, it is *LPCAT2 *up-regulation (and not *LPCAT1*), which is primarily responsible for assisting in PDAT1-catalyzed TAG biosynthesis by supplying an enhanced pool of PC as co-substrate to transfer *sn*-2 moieties to the *sn*-3 position of the enlarged *AS11 *DAG pool. Nonetheless, while LPCAT2 is the most important for maintaining PDAT1-catalyzed TAG synthesis in the *AS11 *background, the partial over-lapping function of LPCATs 1 and 2 identified in this study require further investigation to determine their relative roles in oil synthesis when DGAT1 is functional in a WT background.

The recent discovery of a phosphatidylcholine: diacylglycerol cholinephosphotransferase (PDCT), encoded by the *Arabidopsis ROD1 *gene, indicates that the issue of DAG-PC interconversion during TAG biosynthesis is considerably more complex than previously thought [35]. The PDCT enzyme catalyzes transfer of the phosphocholine headgroup from PC to DAG and is thought to be a major reaction for the transfer of 18:1 into PC for desaturation by FAD2 and FAD3, as well as enabling the reverse transfer of 18:2 and 18:3 into the TAG synthesis pathway. In the *rod1 *mutant, the accumulation of PUFAs in TAGs is reduced by 40%. However, in *AS11 *where 18:3 is strongly increased, and notably, shows a strong stereospecific shift to the *sn*-3 position of TAGs [6], it is unclear whether there is a strong involvement of PDCT; were it so, one would expect a more even distribution of 18:3 at the *sn*-2 and *sn*-3 positions. However, the strong re-distribution of 18:3 proportions to the *sn*-3 and away from the *sn*-2 position in *AS11 *suggests that the major shift in positional composition has to do with PDAT1 catalysis. Thus there are still many questions to address with respect to the roles *ROD1 *and other PC-related metabolic steps such as CPTase might play in oil synthesis in *Arabidopsis *and in *AS11 *in particular.

## Conclusions

**Figure 16:**
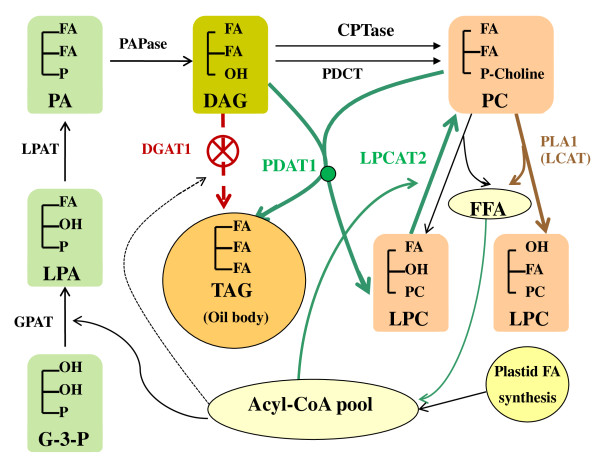
**Schematic showing alterations to the TAG biosynthesis pathway in *AS11 *when DGAT1 activity is absent**. The knockout of DGAT1 is marked in red; enhanced PDAT1 and LPCAT2 are highlighted by green arrows.

Based on the cumulative results of these studies, in Figure 16 we summarize our current hypothesis regarding the TAG biosynthesis pathway in WT *vs*. that observed when DGAT1 is compromised as in *AS11*. When both DGAT1 and PDAT1 are operating they both contribute to TAG synthesis, but the consensus of recent literature supports PDAT1 as a major route in *Arabidopsis*. When DGAT1 is eliminated, the PDAT1 pathway produces up to 75% of WT oil levels [17]. PDAT1 acylates the *sn*-3 position of DAGs which are accumulating (in the absence of DGAT1), to give TAG. The acyl composition of *AS11 *with highly enhanced PUFAs and reduced VLCFAs at the *sn*-3 position [6] supports the donation of acyl groups, especially 18:3, from the *sn*-2 position of PC to the *sn*-3 position of DAG as the major route for TAG synthesis in *Arabidopsis*.

This adjustment in the TAG assembly route in *AS11 *is aided by enhanced LPCAT2 activity to supply the additional PC required by PDAT1. Given these findings and the implicit plasticity of *Arabidopsis *TAG assembly mechanisms to overcome critical bottlenecks, it will be interesting to determine the relative roles played by DGAT1 and [PDAT1 + LPCAT2] during TAG assembly in higher oilseeds (e.g. canola, soybean, sunflower, safflower, flax).

## Methods

### Plant materials and growth conditions

*Arabidopsis *lines, WT (ecotype Columbia-0), and *AS11 *(CS3861) EMS-induced mutant, and *A7 *(SALK_039456), *lpcat1 *(SALK_123480) and *lpcat2 *(SAIL_357_H01) T-DNA insertion mutant lines (all in a Columbia background) were obtained from the Salk Institute via the Nottingham Arabidopsis Stock Centre (University of Nottingham, UK). Seeds of these lines and progeny from genetic crosses were grown in a growth chamber at 22°C with photoperiod of 16 h light (120 μE·m^-2^·s^-1^) and 8 h dark.

Arabidopsis siliques containing mid-green developing embryos (pooled siliques at stages 6- 8, as designated on the University of Toronto Arabidopsis eFP Expression Browser at: http://bar.utoronto.ca/affydb/cgi-bin/affy_db_exprss_browser_in.cgi; see e.g. seed staging in *LPCAT2 *developmental map at http://bbc.botany.utoronto.ca/efp/cgi-bin/efpWeb.cgi?primaryGene=AT1G63050&modeInput=Absolute), were harvested and frozen at -80°C for lipid analyses, enzyme assays and DNA and RNA extraction.

### Lipid analyses

Preparation of total lipid extracts (TLEs) and study of lipid classes, determination of oil content and acyl composition in seeds of WT and the *AS11*, *lpcat1 *and *lpcat 2 *mutant lines and progeny from crosses were performed as described previously [26,36]. In all cases, the data represent the averages of three-five determinations.

### Preparation of Arabidopsis protein fractions

In general, enzyme preparations were made from 200 *Arabidopsis *siliques of *AS11 *and WT containing mid-green developing seeds (stages 7-8 as defined above), and immediately powdered with liquid nitrogen in a mortar and pestle. Grinding medium (100 mM HEPES-KOH, pH 7.4 containing 0.32 M Sucrose, 1 mM EDTA, and 1 mM dithiothreitol; 8 mL/50 siliques) along with 65 mg polyvinylpolypyrrolidone was immediately added, and grinding continued on ice for 5 min. The slurried cell-free homogenate was filtered through two layers of Miracloth (Calbiochem, La Jolla, CA), centrifuged at 3,000 × *g *for 5 min, the pellet discarded and the supernatant re-centrifuged at 15,000 × *g *for 30 min. The supernatant was re-centrifuged at 100,000 × *g *for 1 h and the resultant pellet was resuspended in 2 mL of grinding medium, probe-sonicated on ice for 30 s and protein concentrations were determined using BioRad™ reagent based on the method of Bradford [37]. Protein concentrations were normalized to the same value for WT and *AS11 *in each experiment.

### Assay of TAG synthesis activity in Arabidopsis mid-developing seeds

TAG assembly assays were conducted at pH 7.4, with shaking at 100 rev/min in a water bath at 30°C for 60 min. Assay mixtures (500 μL final volume) contained 100-300 μg protein normalized as described above, 90 mM HEPES-NaOH, 0.5 mM ATP, 0.5 mM CoASH, 1 mM MgCl_2 _in the presence of 100 μM [1-^14 ^C] *sn*-1,2 diolein in 0.02% Tween-20 (specific activity 10 nCi/nmol; pre-purified by TLC on 5% borate silica G plates) and 18 μM unlabeled 18:1-CoA. Reactions were stopped using Isopropanol:CH_2_Cl_2 _(2:1:) v/v, and the TLE prepared as described previously [26,36]. The ^14 ^C-labeled products were resolved by TLC on silica gel G plates developed in hexane: diethyl ether: acetic acid (70:30:1 v/v/v), the ^14 ^C-triolein band visualized on a Bioscan AR-2000 radio-TLC scanner using Win-Scan 2D^© ^software (Bioscan Inc., Washington DC) and the band scraped and quantified on a scintillation counter.

### Expression of mutated AtDGAT1 in yeast

The mutated *AtDGAT1 *(from *AS11 *as well as *A7) *in pYES2.1/NT B plasmid were transformed into a quadruple yeast mutant *H1246MATα *[27] using the *S.c*. EasyComp™Transformation Kit (Invitrogen, San Francisco, CA). Yeast cells transformed with pYES2.1/NT B plasmid containing the WT *DGAT1 *or with empty plasmid were used as positive or negative controls, respectively. Transformants were selected by growth on synthetic complete medium lacking uracil (SC-ura), supplemented with 2% (w/v) glucose. The colonies were transferred into liquid SC-ura with 2% (w/v) glucose and grown at 30°C overnight. The overnight culture was diluted to an OD 0.4 in induction medium (SC-ura + 2% Galactose + 1% Raffinose), and were induced for 24 h at 30°C. The yeast cells were collected and broken with glass beads using a Beadbeater™. The protein concentrations of the yeast cell lysates were normalized using the Biorad™ assay and assayed for DGAT activity. DGAT assays were conducted at pH 7.4, with shaking at 100 rev/min in a water bath at 30°C for 60 min. Assay mixtures (500 μL final volume) contained 100 μg of lysate protein, 90 mM HEPES-NaOH, 100 μM *sn*-1,2 diolein or *sn*-1,2 dierucin (pre-purified by TLC on 10% borate silica H plates and emulsified in 0.02% Tween-20), and 18 μM 1-^14 ^C 18:1-CoA (specific activity 10 nCi/nmol) as the acyl donor. Reactions were stopped using isopropanol:CH_2_Cl_2 _(2:1:) v/v, and extraction of the lipid fraction performed as described previously [26,36]. The ^14 ^C-labeled TAGs were resolved by TLC on silica gel G plates developed in hexane: diethyl ether: acetic acid (70:30:1 v/v/v), the ^14 ^C-triolein band visualized on a Bioscan AR-2000 radio-TLC scanner using Win-Scan 2D^© ^software (Bioscan Inc., Washington DC) and the band scraped and quantified on a scintillation counter.

### Immunodetection

The yeast cell lysates were run on a 10% Tris-HCl SDS-PAGE gel, the proteins were then transferred to a nitrocellulose membrane (Nitrobind, Thermo Fisher Scientific, Waltham, MA). The membrane was blocked in PBST (phosphate buffered saline containing 0.5% Tween 20) containing 4% skim milk for 60 min, and then incubated with the primary antibody, Anti-Xpress (epitope-tagged) antibody (Invitrogen, San Francisco, CA) diluted to 1:5000 with PBST containing 2% skim milk, for 60 min. The membrane was submitted to three washes with PBST followed by three washes with PBS to remove any unbound antibody. Next, the membrane was incubated with a goat anti-mouse IgG peroxidase antibody (Sigma-Aldrich, A2554, St. Louis, MO), diluted to 1:5000 with PBST containing 2% skim milk, for 60 min. The membrane was washed three times with PBST followed by three times with PBS, then the proteins were detected using the Amersham ECL Plus Western Blotting Detection Kit (GE Healthcare Life Sciences) with a 10 min exposure time.

### LPCAT assays

LPCAT assays were conducted at pH 7.4, with shaking at 100 rev/min in a water bath at 30°C in a 20-minute time course. Assay mixtures (500 μL final volume) contained 300 μg protein normalized as described above, 90 mM HEPES-NaOH, 0.5 mM ATP, 0.5 mM CoASH, 1 mM MgCl_2 _in the presence of 6 μM L-α-palmitoyl- [1- ^3^H methyl] *lyso*-3-phosphatidylcholine (specific activity 60 μCi/nmol; 1.48-2.22 TBq/mmol) and 18 μM [1-^14 ^C] 18:1-CoA (specific activity 10 nCi/nmol; 0.37 GBq/mmol). Reactions were stopped at each time point by adding isopropanol: CH_2_Cl_2 _(2:1:) v/v, and the TLE prepared as described previously [26,36]. The ^14 ^C and 3H-labeled products were resolved by 3D TLC performed as follows: The first 2 dimensions were run as described by Yokoyama et al. [38]; 1st D: CHCl_3_: MeOH: Formic Acid (88%):H_2_O 60: 30: 9 : 2; 2nd D: CHCl_3_: MeOH: Ammonia solution (28%): H_2_O 50: 40: 7: 3; 3D: The 3 rd D: 100% ethyl ether. Radiolabeled spots corresponding to standards of LPC and PC were identified using a radio-TLC scanner and the bands scraped and counted on a scintillation counter with a dual ^3^H/^14 ^C isotope measurement program.

### In vivo feeding experiments

Twenty bolted stems with mid-developing siliques of both WT and AS11 plants were harvested under water and then immediately placed in a solution containing 1 μCi of ^14 ^C sodium acetate in 100 μL of water and incubated at room temperature in a fume hood. Once most of this solution was taken up, the plants were supplied with equal aliquots of distilled water during the chase period. At 0, 7, 24 and 30 h, 5 bolted stems were harvested, siliques counted and weighed and then ground with a polytron and a TLE extraction performed as described by Taylor et al. [26]. The lipid extract was resuspended in 1 mL CHCl_3_:MeOH 2:1. 10 μL of the solution was counted and the remainder spotted on TLC plates, 3D TLC performed, radiolabeled spots resolved on a radio-TLC scanner, silica bands scraped and radioactivity measured with a scintillation counter.

### qRT-PCR comparison of AS11 and WT expression of key lipid genes

Triplicate biological samples of total RNA were extracted using the method of Wang and Vodkin [39] from *Arabidopsis *siliques of *AS11 *and WT containing mid-green developing seeds (stages 7-8 as defined above) and used for qRT-PCR [40]. One microgram of total RNA was reverse-transcribed using SuperScript^® ^II Reverse Transcriptase (Invitrogen) according to the manufacturer's protocol. The resulting cDNA was then amplified by PCR using *Taq *DNA polymerase (Invitrogen). PCR conditions comprised an initial cycle of 94°C for 3 min; followed by 30 cycles of 94°C for 30 s, 56°C for 30 s, and 72°C for 1 min; then 72°C for 10 min to complete the reaction. Concentrations for the individual samples within each group (AS11 and WT) were normalized using 18S rRNA levels. The gene-specific primers (listed in Additional file 2: Table S1) were designed according to the target gene sequences as annotated in Genbank™ (http://www.ncbi.nlm.nih.gov/genbank/). Amplified RT-PCR products were resolved by electrophoresis on 1% agarose gels and gel photos taken. The densities of the PCR bands were analyzed using ImageJ image analysis software (National Institutes of Health, USA http://rsbweb.nih.gov/nih-image/) and quantified relative to the "housekeeping gene" 18S rRNA transcript signal. Then the ratio of *AS11*:WT gene expression level was calculated. Specifically, for each gene of interest, first we calculated the ratio of AS11 density/18S rRNA density (= "AS11 ratio") and WT density/18S rRNA density (= "WT ratio"). The difference in specific gene expression in AS11 *vs*. WT, was then calculated as ("AS11 ratio")/("WT ratio") = V. If V >1.0, then the gene is *up-regulated *in *AS11 *relative to WT; if V <1.0, then the gene is down-regulated in *AS11 *relative to WT. The % relative increase or decrease is then = ([**V **× 100]-100).

### Affymetrix microarray analysis

The same triplicate biological samples prepared for the qRT-PCR study (above) were sub-sampled for microarray analysis. Affymetrix microarray hybridizations using the Ath1 whole genome array, containing probe sets representing ~22,800 genes, were performed using three biological replicate samples for each genotype. Labeling, hybridization, and scanning were performed by the Affymetrix Gene Chip Facility at the University of Toronto (http://www.csb.utoronto.ca/resources/facilities/affymetrix-genechip). Data analysis was performed using GeneSpring™ software version 7.2. To identify key lipid genes that were differentially expressed between the two genotypes, a per-gene normalization was applied to the values and a parametric test was performed. Genes that exhibited a false discovery rate of *p *< 0.05 and passed the minimum signal and fold-change threshold were determined to be differentially expressed. Reported candidate genes were selected by comparing those listed by Beisson et al. [33] with our microarray and qRT-PCR data. The "fold change" was converted to % change. For the group of genes detailed in Figure 3, the final changes ranged from -60% to + 150%.

## Mutant crosses

### Confirmation of lpcat1 and lpcat2 T-DNA insertion mutation lines

*Arabidopsis *insertion mutation lines [41] SALK_123480 putative for *LPCAT1 *(At1g12640) and SAIL_357_H01 putative for *LPCAT2 *(At1g63050) were identified in the Salk Institute T-DNA insertion library database (http://signal.salk.edu/cgi-bin/tdnaexpress), and seeds were obtained from the Nottingham Arabidopsis Stock Centre (University of Nottingham, UK). According to annotation in the database SALK_123480 contains a T-DNA insertion in the middle of the 7th exon of the *LPCAT1 *gene and SAIL_357_H01 a T-DNA insertion in the 8th exon of the *LPCAT2 *gene. Individual plants homozygous for a T-DNA insertion in each the *LPCAT1 *or *LPCAT2 *genes were identified by PCR screening using primers SALK_123480LP, SALK_123480RP and SALK_LBb1 (Additional file 2: Table S2) for *LPCAT1 *and SAIL_357_H01LP, SAIL_357_H01RP and SAIL_LB2 (Additional file 2: Table S2) for *LPCAT2*. Individual plants from each mutant line lacking a T-DNA insertion in the *LPCAT1 *or *LPCAT2 *genes (null segregants) were also identified in the same PCR primer set. Annotation of lines from each set of crosses is as designated in Additional file 2: Table S3.

### Transcript analyses of lpcat1, lpcat2, dgat1 mutant lines and crosses

#### lpcat1 (See Figure 4)

Total RNA was isolated from developing siliques from control and SALK_12340 T-DNA insertion mutation plants using CONCERT Plant RNA Reagent (Invitrogen, San Francisco, CA) according to the technical specifications given by the manufacturer. The RNA was then DNAse-treated with TURBO™ DNase (Ambion, Huntingdon, UK)) and used (1 mg) for synthesize first strand cDNA with Maxima First Strand cDNA Synthesis Kit (Fermentas.Thermo Fisher Scientific, St. Leon-Rot, Germany). The cDNA was amplified by PCR using Taq DNA Polymerase (Sigma-Aldrich, St. Louis, MO) and the primers SALK_12640-1 s and SALK_12640-6r as listed in Additional file 2: Table S2. PCR conditions comprised an initial cycle of 94°C for 1 min; followed by 40 cycles of 94°C for 20 s, 64°C for 20 s, and 72°C for 35 s; then 72°C for 8 min to complete the reaction. PCR samples were separated on 1% agarose gel using λ-DNA digested with PstI as size marker.

#### lpcat2 and AS11 dgat1 (See Figure 5)

Total RNA was isolated from developing siliques from WT control, *AS11*(*dgat1*), SAIL_357_H01 insertion mutation (*lpcat2*) and AS11/l*pcat2 *plants by the method of Wang and Vodkin [39]. One microgram of total RNA was reverse-transcribed using SuperScript^® ^II Reverse Transcriptase (Invitrogen) according to the manufacturer's protocol. The resulting cDNA was then amplified by PCR using *Taq *DNA polymerase (Invitrogen). PCR conditions comprised an initial cycle of 94°C for 3 min; followed by 30 cycles of 94°C for 30 s, 56°C for 30 s, and 72°C for 1 min; then 72°C for 10 min to complete the reaction. Concentrations for the individual samples within each group (AS11 and WT) were normalized using 18S rRNA levels. Gene specific primers spanning the T-DNA insertional region were used to check for gene transcription. For *DGAT1 *transcript analysis in lines of WT, the *AS11 *(*dgat1*) mutant, *lpcat2 *mutant and crosses of *lpcat2 *with *dgat1*, the primer pairs were TAG1-mut-primerA and TAG1-mut-primerB. For *LPCAT2 *transcript analysis in these same lines, the primer pairs were LPCAT2-F2 and LPCAT2-R2 as listed in Additional file 2: Table S2.

### Creating double mutants of dgat1 × lpcat1 and dgat1 × lpcat2

Crosses between the *AS11 *mutant and *lpcat1 *and *lpcat2 *T-DNA insertion mutation lines, respectively, were made and F_1 _plants heterozygous for *AS11 *and the insertion mutations were identified by PCR using primers listed in Additional file 2: Table S2. F_2 _seeds segregating for the mutations were planted and screened by PCR. All PCR screening was done as above for *LPCAT1 *and *LPCAT2 *and for the *AS11 *mutation *vs *WT using primers dgat1-mut-primerA and dgat1-mut-primerB (Additional file 2: Table S2), as designed by Zou et al. [7]. Identification of individuals homozygous for both the *AS11 *mutation and *lpcat2 *insertion mutation was only possible after growing a segregating F_2 _seed population (homozygous for *AS11 *and heterozygous for *lpcat2 *mutant) on agar media containing a 1/3 strength MS and 1% sucrose (Additional file 2: Table 3).

## Authors' contributions

JX performed the qRT-PCR of lipid gene expression in *AS11 *and WT and assisted in the design of this study, ASC carried out the crosses of the *lpcat *mutants with *AS11 *and selection of the lines with heterozygous or homozygous mutation conditions, TF carried out the microarray analyses of AS11 and WT gene sets and the ectopic expression of the mutated AS11 and A7 DGAT1 proteins in yeast mutant *H1246MATα*, MZ carried out the work demonstrating the dominant role of PDAT1 in *Arabidopsis *TAG assembly and provided advice regarding the analyses of the mutant crosses, TH and MG performed the LPCAT and DGAT assays as well as the oil content, seed weight and acyl profiling of the mutant crosses. DCT conceived of the study, led its design and coordination and drafted the manuscript. All authors read and approved the final manuscript.

## Supplementary Material

Additional file 1 **Figure S1 **Comparison of growth phenotypes of WT, *AS11 *and *A7 Arabidopsis thaliana *lines. While the WT has bolted, both *AS11 *and *A7 *show a delay in plant development: the generative (reproductive) phase is delayed about 1 week (33% longer than WT to mature). Figure S2. (**A**) Photos of developing siliques from F_2 _*dgat1*/*lpcat2 *He/H lines #6-3-7 (left) and # 6-3-10 (right) showing the many gaps due to non-fertilized ovules. (**B**) Photos 1-4: Close-ups of developing siliques of line #6-3-10 with arrows pointing to non-fertilized ovules (photos 1 & 2). Figure S3. Photos of developing siliques from F_2 _*dgat1*/*lpcat2 *H/He line #6-3-13 showing normal seed development and pattern. Click here for file

Additional file 2 **Table S1 **Primers used for semi-quantitative RT-PCR conducted in this study. Table S2. Primers used to screen the *AS11 dgat1 *EMS mutant and *lpcat1 *and *lpcat2 *T-DNA insertion mutant lines by PCR. Table S3. Details on crosses performed to develop lines homozygous for both *dgat1 *and *lpcat1 *mutations or for both *dgat1 *and *lpcat2 *mutations. Click here for file
