# Supplementary figures and images for: Triacylglycerol synthesis by PDAT1 in the absence of DGAT1 activity is dependent on re-acylation of LPC by LPCAT2
Source: BMC Plant Biol. 2012 Jan 10;12:4. doi: 10.1186/1471-2229-12-4 (PMC3310826; doi:10.1186/1471-2229-12-4)

## Slide 1
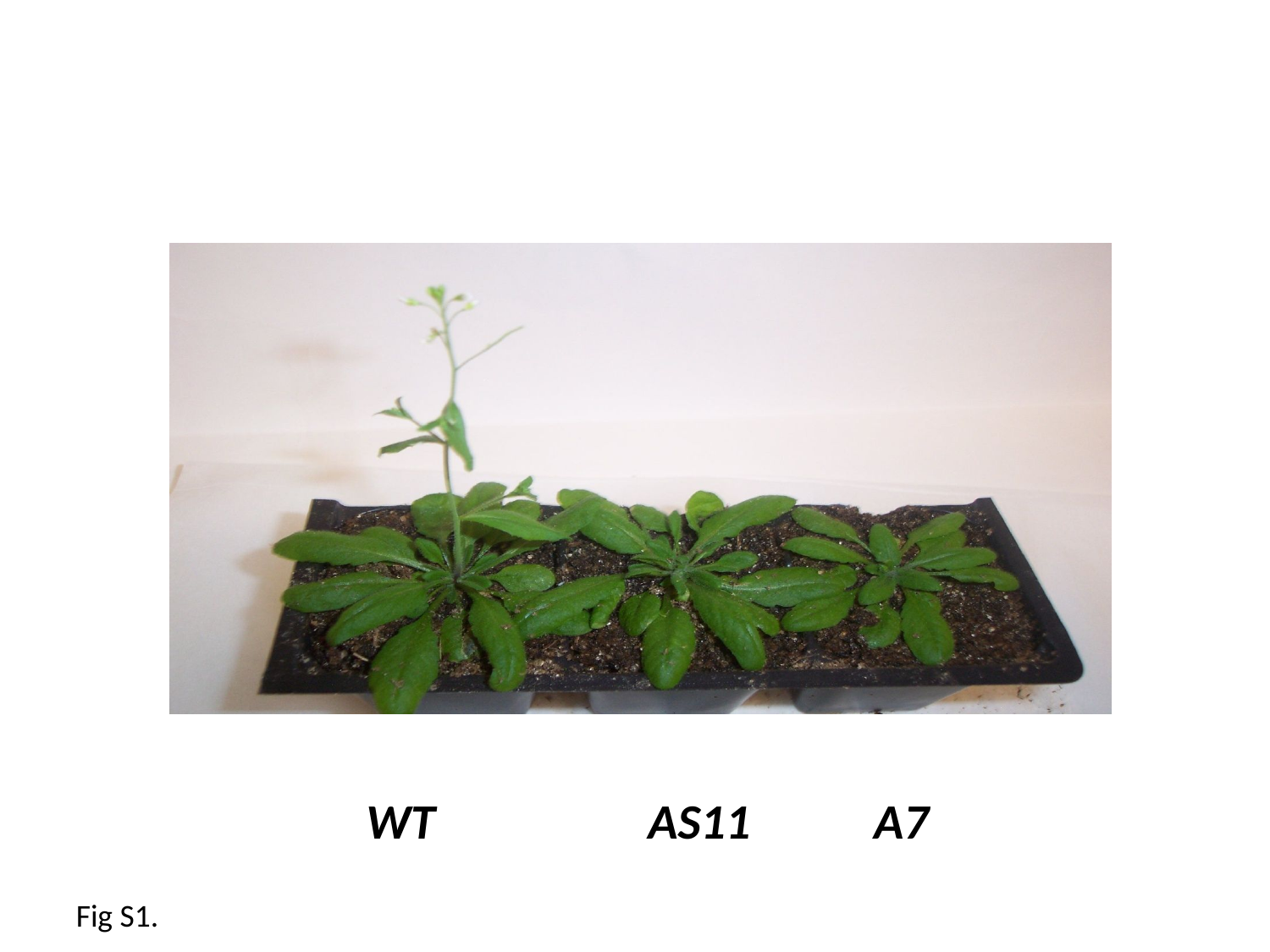

WT AS11 A7
Fig S1.

## Slide 2
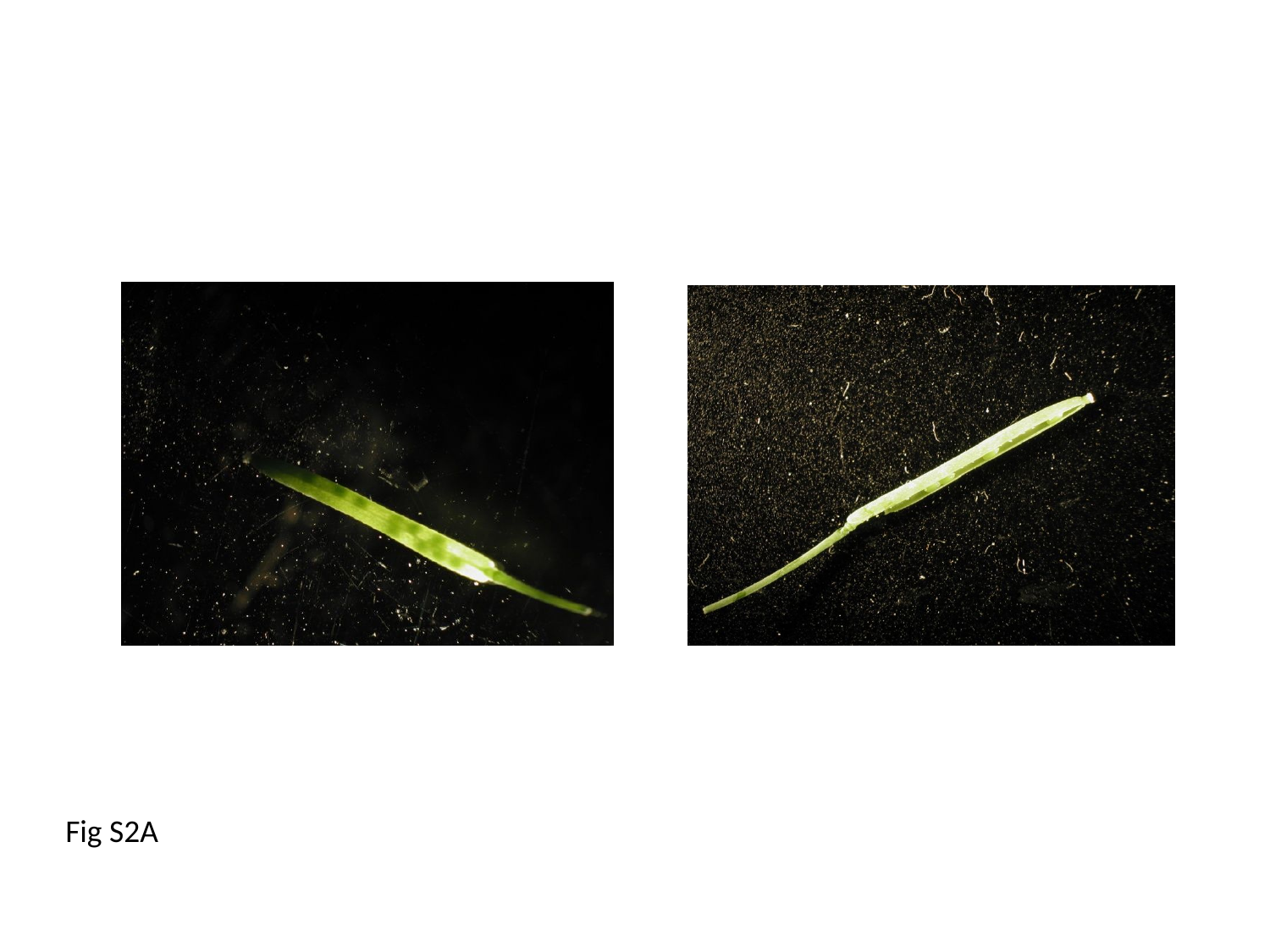

Fig S2A

## Slide 3
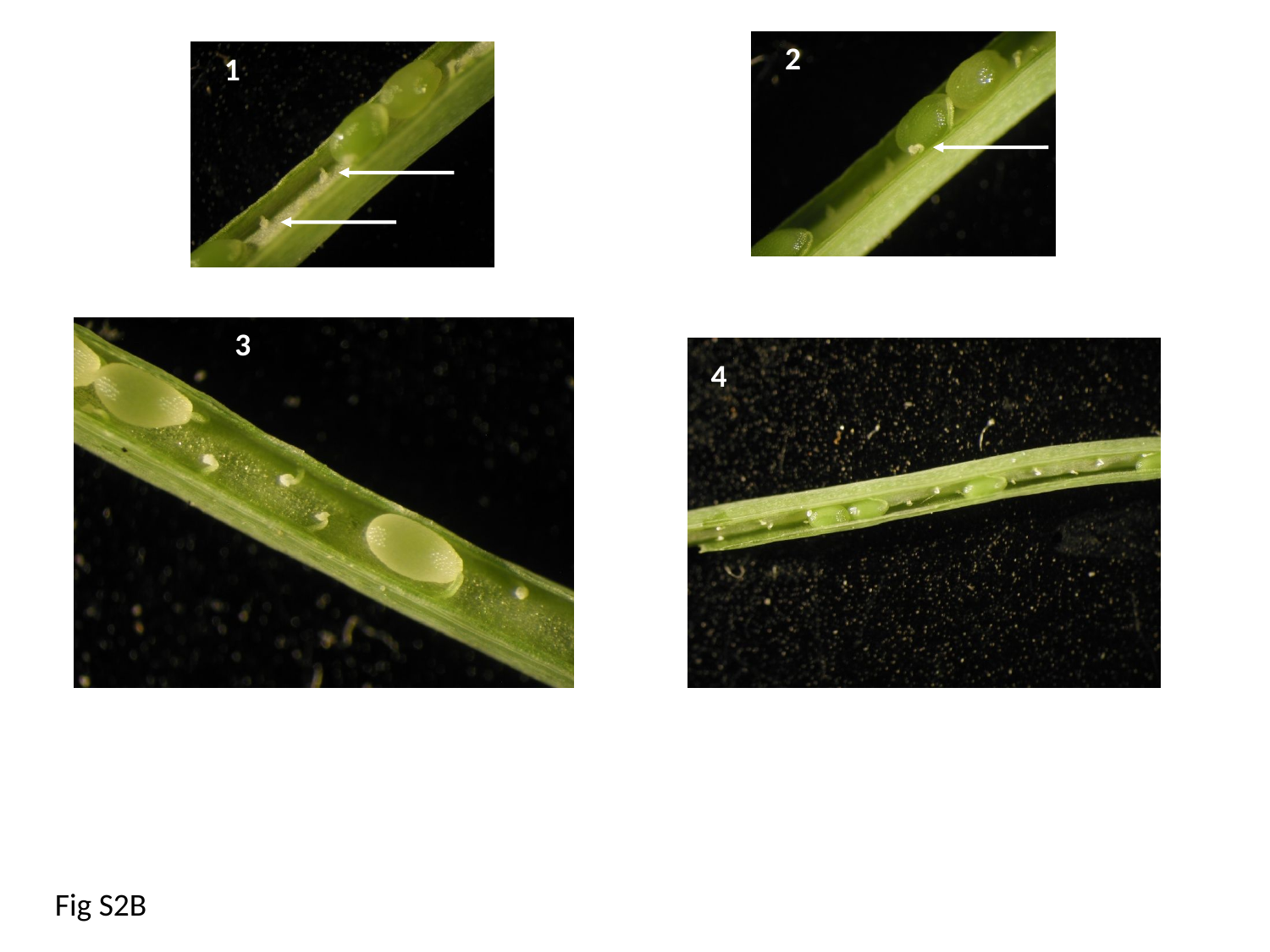

2
1
3
4
Fig S2B

## Slide 4
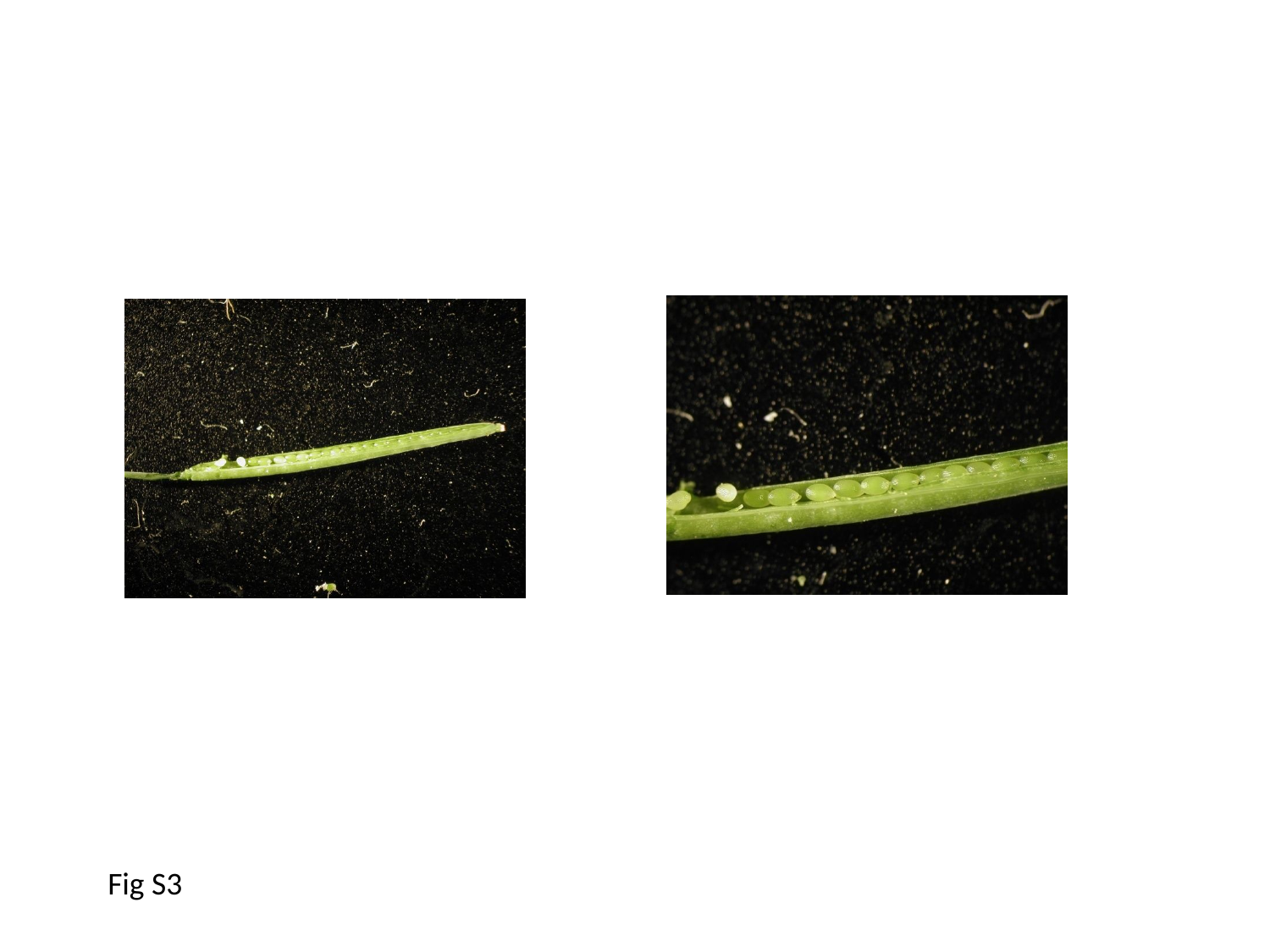

Fig S3

Supplement: Additional file 1 — Figure S1 Comparison of growth phenotypes of WT, AS11 and A7 Arabidopsis thaliana lines. While the WT has bolted, both AS11 and A7 show a delay in plant development: the generative (reproductive) phase is delayed about 1 week (33% longer than WT to mature). Figure S2. (A) Photos of developing siliques from F2 dgat1/lpcat2 He/H lines #6-3-7 (left) and # 6-3-10 (right) showing the many gaps due to non-fertilized ovules. (B) Photos 1-4: Close-ups of developing siliques of line #6-3-10 with arrows pointing to non-fertilized ovules (photos 1 & 2). Figure S3. Photos of developing siliques from F2 dgat1/lpcat2 H/He line #6-3-13 showing normal seed development and pattern. [file 1471-2229-12-4-S1.PPT]
