# Supplementary material for: Triacylglycerol synthesis by PDAT1 in the absence of DGAT1 activity is dependent on re-acylation of LPC by LPCAT2
Source: BMC Plant Biol. 2012 Jan 10;12:4. doi: 10.1186/1471-2229-12-4 (PMC3310826; doi:10.1186/1471-2229-12-4)
Supplement: Additional file 2 — Table S1 Primers used for semi-quantitative RT-PCR conducted in this study. Table S2. Primers used to screen the AS11 dgat1 EMS mutant and lpcat1 and lpcat2 T-DNA insertion mutant lines by PCR. Table S3. Details on crosses performed to develop lines homozygous for both dgat1 and lpcat1 mutations or for both dgat1 and lpcat2 mutations. [file 1471-2229-12-4-S2.PDF]

Table S1. Primers used for semi-quantitative RT-PCR conducted in this study.

Gene name	AGI code	Forward primer	Reverse primer
18S	At2g47990	5'-GGTAGGCGATTGGCTAAATTGTCTGC-3'	5'-GAGACACCAACAGTCTTTCCTCTGCG-3'
LPCAT1	At1g12640	5'-GGGACCGTGCCAAGAACGTAGAT-3'	5'-CAAGCGCGGGAAACTTACTTACCG-3'
LPCAT2	At1g63050	5'-TGGCCCGGTTTTCGAAATGAAAG-3'	5'-AGCATTGCCATTTTCGGAGGTATTG-3'
DGAT1	At2g19450	5'-TTGGATTCTGCTGGCGTTACTACG-3'	5'-TCTCGCAGCGATCTTGAACTAAACC-3'
DGAT2	At3g51520	5'-GCTTTCCAGCCTAATCGTGCCTATG-3'	5'-CTGTCGACACGGTAATGGTGATCCA-3'
FAD2	At3g12120	5'-TAACGTTATCGCCCCTACGTCAGC-3'	5'-AATTGGTGGCGACGTAGTAGAAGCA-3'
FAD3	At2g29980	5'-CACTCGCGGTTCTTAAAGTCTACGG-3'	5'-CCACCAAGTGGATCGGTATTGCTC-3'
FAE1	At4g34250	5'-TCTACTAATAATCCTTGGGAACAG-3'	5'-AAGTCCACTACCACGCATCAAA-3'
PDAT1	At5g13640	5'-GCGATTGCCCCAGGATTCTTAGAC-3'	5'-CCACGCGCCATCATCTTAGGAG-3'
PDAT2	At3g44830	5'-ACCCGCAAAGTACGGAAGAATCG-3'	5'-GGTCGGAATCCCTACTCCGTAAAGA-3'

Table S2. Primers used to screen EMS mutant and T-DNA insertion mutations lines by PCR and RT-PCR

Gene name	AGI code	Mutant ID	Primer name	Sequence
DGAT1	At2g19450	AS11 (CS3861)	TAG1-mut-primerA	5'-CGACCGTCGGTTCCAGCTCATCGG-3'
			TAG1-mut-primerB	5'-GCGGCCAATCTCGCAGCGATCTTG-3'
LPCAT1	At1g12640	SALK_123480	SALK_123480LP	5'-CCAATACATGGCTGGATTCAC-3'
			SALK_123480RP	5'-AAGCGCGGGAAACTTACTTAC-3'
			SALK_12640-1s	5'- GTGTAAAGGAAGTTGATGAAGACC -3'
			SALK_12640-6r	5'- GAGTTCAATGGCTGGTTCAATCG -3'
LPCAT2	At1g63050	SAIL_357_H01	SAIL_357_H01LP	5'-TTTGGTTACCAATACATGGCG-3'
			SAIL_357_H01RP	5'-GAAGGCGACTAGTGTTTCGTG-3'
			LPCAT2-F2	5'-GGTTTGGGTTTCAGTGGTTGGACTG-3'
			LPCAT2-R2	5'-TTGGTCTAACAGGCTTCACAGGAAC-3'
Binds near the left border of T-DNA			SALK_LB1	5'-GCGTGGACCGCTTGCTGCAACT3'
Binds near the left border of T-DNA			SAIL_LB2	5'-GCTTCCTATTATATCTTCCCAAATTACCAATACA-3'

Table S3. Details on crossing work to develop lines homozygous for both *dgat1* and *lpcat1* or for both *dgat1* and *lpcat2* mutations.

**Single mutation lines**	**AGI code**	**Mutant ID**	**Comments**
dgat1	At2g19450	AS11 /CS3861	
lpcat1	At1g12640	N123480-6	
lpcat2	At1g63050	CS816604-9	
**Double mutation lines**			
dgat1* X *lpcat1		CS3861 x N123480-6	Crosses made and F_1 seeds harvested
CS3861 x N123480-6 #3-3			DNA checked on this F_1 individual and confirmed hemizygous for both genes
CS3861 x N123480-6 #3-3-12<br><br>CS3861 x N123480-6 #3-3-9			F_2 individuals identified as homozygous for mutations in both genes (*dgat1* and *lpcat1* (#3-3-12) and null for mutations in both (#3-3-9)
dgat1* X *lpcat2		CS3861 x CS816604-9	Crosses made and F_1 seeds harvested
CS3861 x CS816604-9 #6-3			DNA checked on this F_1 individual and confirmed hemizygous for mutations in both genes (*dgat1* and *lpcat2*)
CS3861 x CS816604-9 #6-3-7<br>CS3861 x CS816604-9 #6-3-10<br>CS3861 x CS816604-9 #6-3-13<br>CS3861 x CS816604-9 #6-3-20			F_2 individuals identified as hemizygous for a mutation in *dgat1* and homozygous for a mutation in *lpcat2* (#6-3-7and #6-3-10);<br>F_2 individuals identified as homozygous for mutation in *dgat1* and hemizygous for mutation in *lpcat2* (#6-3-13 and #6-3-20)
CS3861 x CS816604-9 #6-3-20-1<br>CS3861 x CS816604-9 #6-3-20-3<br>CS3861 x CS816604-9 #6-3-20-8<br>CS3861 x CS816604-9 #6-3-20-4<br>CS3861 x CS816604-9 #6-3-10-19			Selected additional lines on MS agar plates (1/3 MS + 1% sucrose) homozygous for mutations in both *dgat1* and *lpcat2* (#6-3-20-1, #6-3-20-3 and #6-3-20-8) and null for mutations in both (#6-3-20-4).<br>Re-selected F_3 line homozygous for mutations in both *dgat1* and *lpcat2* (#6-3-10-19)
